# The combination of Hsp90 inhibitor 17AAG and heavy-ion irradiation provides effective tumor control in human lung cancer cells

**DOI:** 10.1002/cam4.377

**Published:** 2015-01-13

**Authors:** Hirokazu Hirakawa, Hiroshi Fujisawa, Aya Masaoka, Miho Noguchi, Ryoichi Hirayama, Momoko Takahashi, Akira Fujimori, Ryuichi Okayasu

**Affiliations:** 1 International Open Laboratory and Research Center for Charged Particle Therapy/Research Center for Radiation Protection, National Institute of Radiological SciencesChiba, 263-8555, Japan; 2 Department of Bioengineering, School of Engineering, The University of TokyoTokyo, 113-8656, Japan; 3 Advanced Science Research Center, Japan Atomic Energy AgencyTokai-mura, 319-1195, Japan; 4 Translational Research Center, Fukushima Medical UniversityFukushima City, 960-1295, Japan

**Keywords:** 17AAG, DNA repair, heavy ions, Hsp90 inhibitor, lung cancer

## Abstract

Hsp90 inhibitors have become well-studied antitumor agents for their selective property against tumors versus normal cells. The combined treatment of Hsp90 inhibitor and conventional photon radiation also showed more effective tumor growth delay than radiation alone. However, little is known regarding the combined treatment of Hsp90 inhibitor and heavy-ion irradiation. In this study, SQ5 human lung tumor cells were used in vitro for clonogenic cell survival and in vivo for tumor growth delay measurement using a mouse xenograft model after 17-allylamino-17-demethoxygeldanamycin (17AAG) pretreatment and carbon ion irradiation. Repair of DNA double strand breaks (DSBs) was also assessed along with expressions of DSB repair-related proteins. Cell cycle analysis after the combined treatment was also performed. The combined treatment of 17AAG and carbon ions revealed a promising treatment option in both in vitro and in vivo studies. One likely cause of this effectiveness was shown to be the inhibition of homologous recombination repair by 17AAG. The more intensified G2 cell cycle delay was also associated with the combined treatment when compared with carbon ion treatment alone. Our findings indicate that the combination of Hsp90 inhibition and heavy-ion irradiation provides a new effective therapeutic alternative for treatment of solid tumors.

## Introduction

Proton and heavy-ion irradiation have become good alternatives to the conventional photon radiotherapy due to fine targeting of tumor tissues against surrounding normal tissues 1–5. In addition to this physical property, so-called high linear energy transfer (LET) heavy ions can provide further biological advantages as a higher rate of tumor cell killing can be obtained when compared with conventional X- or gamma-rays 2,4–8. Mouse tumor studies with carbon ions from our institute showed significantly high relative biological effectiveness (RBE) as compared with gamma-rays 9. Recently our group also showed that tumor stem-like cells might be better controlled by carbon ion beams than X-rays 10. Although heavy-ion treatment has been successful and the local tumor control rate is generally high (over 90% in some cases), it has not reached 100% and the patient survival rates are much lower in general 3,4. Therefore, further improvement of carbon ion therapy would be necessary. One of the ways to improve the tumor cure rate as well as overall patient survival would be to combine carbon therapy with other therapeutic modalities 2. In this report, we are proposing the combined treatment with an Hsp90 inhibitor and carbon ion irradiation; our study includes a mouse model with human tumor cells.

Hsp90 is a molecular chaperone protein abundantly present in cells, and its inhibition has been extensively exploited in recent years for its antitumor effect 11–13. As Hsp90 is known to be essential for malignant transformation and progression 13,14, inhibiting this molecule would be a good strategy for tumor control. A cause of tumor selective properties of Hsp90 inhibitors has been described 15. Although there is no FDA-approved Hsp90 inhibitor 16, 17 agents have entered clinical trials 12,17. The combination of Hsp90 inhibitor and radiation on tumor cells has been studied and enhancement of radiation effect with the inhibitors has been well documented 18–30. Hypoxic tumor cells were also radiosensitized by the combination strategy 31,32. Some of these studies indicated that, as compared with tumor cells, normal cells might not be affected, indicating a selective radiosensitization of tumor cells 18,19,21. We and others have also shown that one of the causes of sensitization could be inhibition of DNA double strand break (DSB) repair 21–23,25–27. Checkpoint arrest mainly at G2/M phase has also been suggested as a cause of radiosensitization with Hsp90 inhibitors 23–29,32. An interesting study recently reported that Hsp90 inhibitor 17AAG induces BRCA1 ubiquitination and proteasomal degradation, leading to repair inhibition of DSBs induced by ionizing radiation 33. Radiosensitization effect in vivo by Hsp90 inhibitors has also been demonstrated 18,26,28.

As we showed evidence that 17AAG affected the homologous recombination (HR) pathway of DNA DSB repair when combined with low LET X-rays 21, and one recent report indicated that the combination of heavy ions with targeting HR pathway by microRNAs yielded a radiosensitizing effect 34, we wanted to test whether 17AAG enhances the effect of high LET heavy ions in tumor cells. Our in vitro and in vivo results seemed to indicate that the combination of Hsp90 inhibitor 17AAG and carbon ion irradiation provides better tumor control than carbon irradiation alone.

## Materials and Methods

### Cell culture, drug treatment and irradiation

Human lung squamous carcinoma cell line SQ-5 was obtained from RIKEN Bio Resource Center and was grown in *α*-MEM supplemented with 10% FBS (Fetal bovine serum) and antibiotics. Normal human embryonic lung fibroblasts HFL III were obtained from RIKEN Bio Resource Center and grown in *α*-MEM supplemented with 15% FBS and antibiotics. 17AAG (Wako, Osaka, Japan) was dissolved in dimethyl sulfoxide (DMSO) to a stock concentration of 1 mmol/L and stored at −30°C. Cells were irradiated with a Shimadzu (Koto-ku, Tokyo, Japan) Pantak HF-320 X-ray machine at a dose rate of 0.93 Gy/min. Heavy-ion irradiation was performed with the heavy-ion medical accelerator in Chiba (HIMAC) at the National Institute of Radiological Sciences (NIRS), and LET of 70 keV/*μ*m mono-peak irradiation condition or spread-out Bragg peak (SOBP) condition with LET around 50 keV/*μ*m was used for the experiment. These LET values are similar to those used in clinical practice.

### Cell survival assay

Cell survival was measured by colony formation assay. 17AAG or DMSO was added to growth media and incubated for 24 h at 37°C. Then cells were irradiated with X-rays or carbon ions (0–6 Gy), trypsinized, diluted, counted and immediately seeded in 60-mm dishes at various cell densities. After 2 weeks of incubation, colonies were stained with crystal violet, and the colonies containing more than 50 cells were counted. Cell survival experiment was performed at least twice for each modality, and for each data point, three dishes were used. Cell survival values by 17AAG alone (with DMSO) were 60–70% for SQ5 and 80–90% for normal cells.

### DNA DSB repair assay

The method has been reported previously 35. Immediately after irradiation (20 Gy) on ice, the medium was replaced with warm medium and cells were incubated (37°C) for repair. At each repair point, cells were washed, trypsinized on ice for 20 min, and washed again in cold medium. The resulting cell pellet was embedded in 1% agarose (InCert agarose; FMC, Rockland, ME USA) at a density of 1.5 × 10^6^ cells/mL, and placed on ice. These agarose samples were cut into plugs and placed in lysis solution (Trevigen, Gaithersburg, MD USA) containing proteinase K for 1 h on ice, and then incubated for 24 h at 50°C. The plugs were equilibrated in TE buffer (Ph 8.0; Sigma, St. Luis, MO USA), loaded on 0.6% SeaKem Gold agarose gels (Cambrex, Walkersville, MD USA), and subjected to electrophoresis at 0.6 V/cm in 0.5× TBE buffer for 36 h. The gel was stained with ethidium bromide and destained. Fluorescence intensities were measured with an UV transilluminator and a digital camera with an orange filter. NIH Image software (Bethesda, MD USA) was used for the analysis of DSB damage, and the fraction of released DNA was calculated.

### Antibodies

The following primary antibodies were used for immunoblot and immune-staining: mouse monoclonal anti-Ku80 (Ab-2 clone 111), mouse monoclonal anti-Ku70 (Ab-4 clone N3H10), mouse monoclonal anti-DNA-PKcs (Ab-4) (Thermo Scientific, Waltham, MA USA), rabbit polyclonal anti-Rad51 (H-92) (Santa Cruz, Dallas, TX USA), rabbit polyclonal anti-Hsp90 (C45G5), rabbit polyclonal anti-Mre11, rabbit polyclonal anti-GAPDH (14C10) (Cell Signaling Technology Danvers, MA USA), and mouse monoclonal anti-Cyclin B1 (Millipore, Billerica, MA USA). Secondary antibodies used: goat anti-mouse or goat anti-rabbit IgG (H+L)-HRP conjugate (Bio-Rad Laboratories, Hercules, CA USA), Alexa-488-conjugated goat anti-mouse IgG and Alexa-594-conjugated goat anti-rabbit IgG (Molecular Probes, Eugene, OR USA).

### Immunoblot analysis

Whole-cell extracts were prepared as described previously 36. Briefly, cells were washed with ice-cold PBS (Phosphate Buffered saline) and harvested by scraping. Cells were resuspended in Buffer I (10 mmol/L Tris-HCl, pH 7.8, 200 mmol/L KCl, and protease inhibitor cocktail [Roche, Eugene, OR USA]). Then an equal volume of Buffer II (10 mmol/L Tris-HCl, pH7.8, 200 mmol/L KCl, 2 mmol/L KCl, 2 mmol/L EDTA, 40% glycerol, 0.2% NP-40, 2 mmol/L DTT, and protease inhibitor cocktail) was added. Cell lysates were collected after incubation and centrifugation. Protein concentrations were measured by protein assay kit (Bio-Rad Laboratories). Total cellular lysates were loaded onto NuPAGE4-12% Bis-Tris gel (Invitrogen, Carlsbad, CA USA), separated by electrophoresis at a constant voltage (125 V) and electro-transferred onto nitrocellulose membranes at 35 V. Membranes were blocked for 1 h at room temperature in 5% nonfat dry milk in Tris-Buffer saline containing 0.1% (v/v) Tween20 (TBST). The membrane was incubated with primary antibody for 2 h at room temperature. Membranes were washed with TBST and incubated with a secondary antibody for 1 h at room temperature. After washing the membrane with TBST, HRP activity was detected using Western Lightning ECL Pro (PerkinElmer, Waltham, MA USA) and analyzed by GeneGnome imager (Syngene, Cambridge, UK).

### Immunofluorescence measurements

Cells were grown as monolayers on chamber slides with plastic bottom (Nunc Lab-Tek, Roskilde, Denmark), fixed in 4% paraformaldehyde in PBS for 15 min at room temperature, and washed in PBS. Then the cells were permeabilized in 0.5% Triton X-100 for 10 min, and blocked in PBS with 3% BSA (Bovine serum albumin) for 20 min at room temperature. The cells were sequentially incubated with primary antibodies and fluorescence-labeled secondary antibodies for 1 h at 37°C in PBS with 1.5% BSA and washed three times in PBS. Cover glasses were mounted in ProLong Gold Antifade with 4, 6-diamidino-2-phenylindole (DAPI) (Molecular Probes). Fluorescence images were captured with an Olympus (Nishishinjuku, Tokyo, Japan) BX51 epifluorescence microscope with PlanApo 60 (NA = 1.40) lens and a CCD camera.

### In vivo mouse xenograft experiment

BALB/c-nu/nu mice (male, 7 weeks old) were subcutaneously inoculated with a total of 1 × 10^6^ SQ-5 cells in 10 *μ*L of 1× PBS(-) into their right hind legs to prepare the tumor model. Tumors were allowed to grow for 1 week (average volume ∽50 mm^3^). Mice were then treated intraperitoneally three times daily before irradiation with 17AAG (80 mg/kg). A day after final administration of 17AAG, the mice were placed on a Lucite plate and the tumors were irradiated locally at 5, 10, and 20 Gy by 270 MeV/n accelerated carbon ions using SOBP or at 10 Gy by Cs-137 gamma-rays. The size of palpable tumors was measured with a caliper every 2–3 days. Tumor volume (*V*) was estimated by *V* = *π*/6 × length × width × height. Five mice were used per treatment option and the experiment was repeated at least twice. We did not observe any significant systemic effects in mice after 17AAG treatment and/or local carbon ion exposure. All animal experiments were carried out in accordance with the guidelines for animal experiments of NIRS, Japan.

## Results

### Tumor cells were selectively radiosensitized with 17AAG pretreatment after carbon ion irradiation

**Figure 1:**
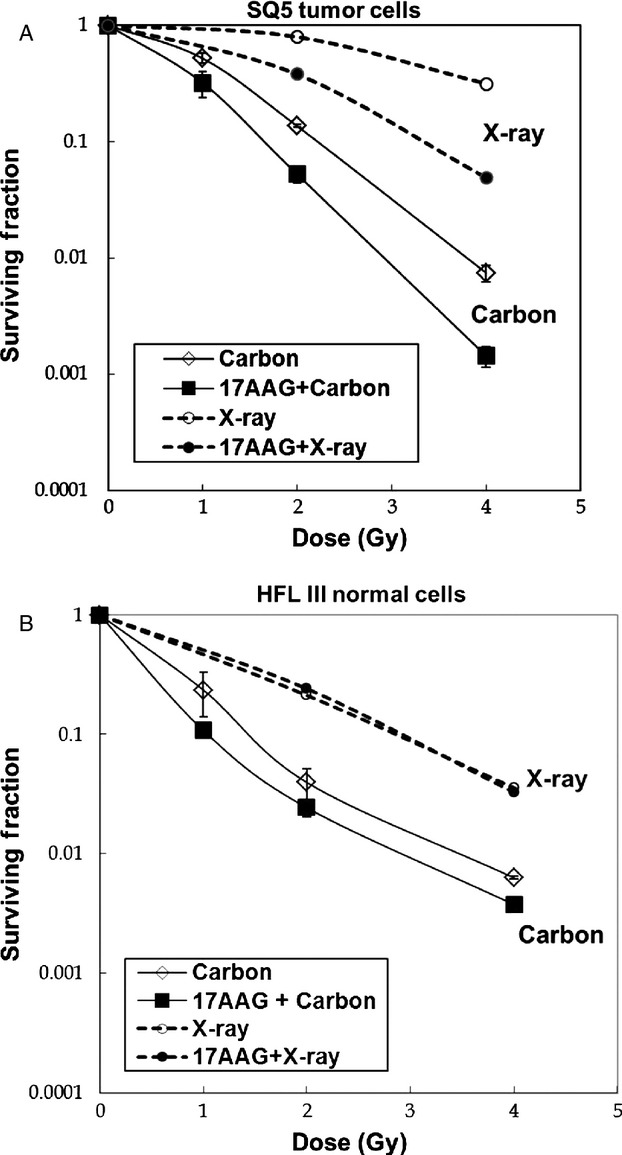
(A) Clonogenic cell survival curves of SQ5 human lung tumor cells irradiated with 70 keV/*μ*m carbon ions (mono-peak) with and without pretreatment of 100 nmol/L 17AAG. Corresponding survival curves with X-rays are shown in dotted lines. (B) Clonogenic survival curves of normal human HFL III cells irradiated with 70 keV/*μ*m carbon ions with and without pretreatment of 100 nmol/L 17AAG. Corresponding cell survival curves with X-rays are shown in dotted lines.

In vitro cell survival was examined in SQ5 lung tumor cells pretreated with 17AAG and irradiated with carbon ions (Fig.1A). For comparison, data with carbon ions alone and results obtained with X-rays 21 are also shown in the Figure. The RBE for 70 keV/*μ*m carbon ions at 0.1 survival fraction is about 2.5 (about 2.0 at 0.01 survival level). As in the case with X-rays, 17AAG significantly radiosensitized SQ5 tumor cells with carbon irradiation. This was a rather unexpected finding as heavy ion itself is known to affect the repair process for reducing cell survival 5. In contrast, 17AAG did not significantly change the cell survival level in exponentially growing HFL III normal lung human cells exposed to carbon ions, suggesting that tumor cells are more selectively influenced by 17AAG than normal cells (Fig.1B). The same trend was also shown previously with X-rays for two tumor cell lines and HFL III cells, and we also have preliminary data showing radiosensitization with carbon ions for at least another tumor cell line 21.

### The combination treatment with 17AAG and local carbon ion irradiation shows effective tumor growth delay in vivo

**Figure 2:**
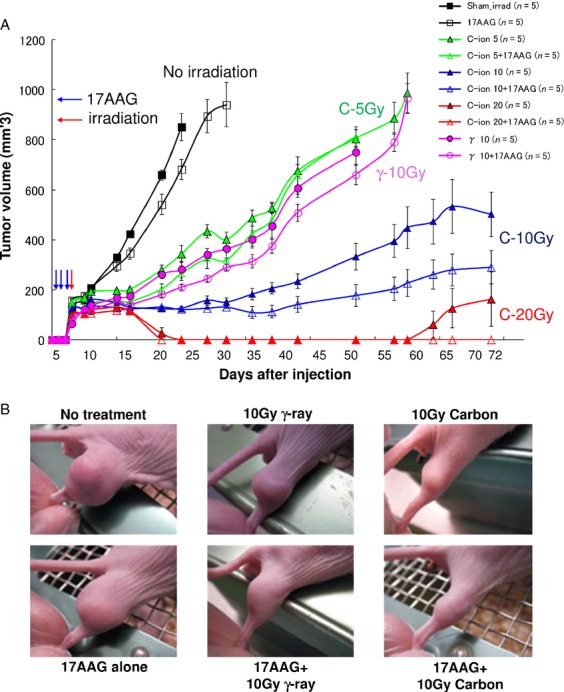
(A) In vivo tumor growth curves by mouse xenograft after various doses of carbon ion and gamma-ray (single 10 Gy dose) irradiation with or without 17AAG pretreatment; radiation was given one time locally using SOBP and 17AAG was given intraperitoneally for three consecutive days before irradiation. Five mice are used for each treatment and error bars represent standard error from five measurements. (B) Representative photographs of tumor growth in mouse xenograft assay (42 days after cell inoculation). SOBP, spread-out Bragg peak.

**Figure 3:**
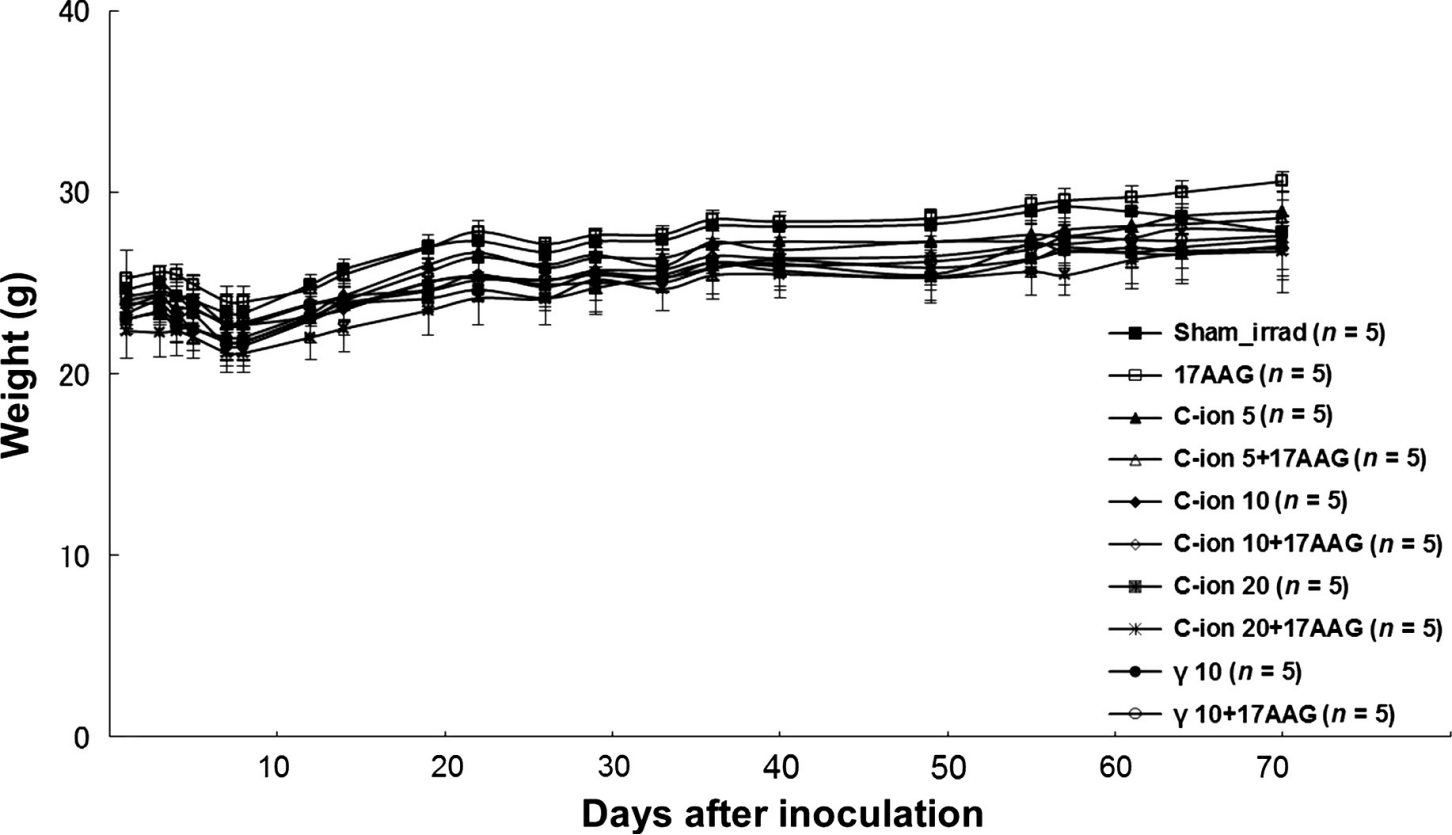
Data of weight measurements of mice after various radiation and 17AAG pretreatments.

In vivo tumor growth curves for various carbon doses with and without 17AAG pretreatment using SQ5 tumor cells implanted in a leg of nude mice are shown in Figure2A. All similar in vivo radiation experiments performed by us earlier showed the same tendency as indicated in this Figure. A clear dose response with local carbon ion treatment can be observed. A tumor growth curve with gamma-irradiated sample is also provided for comparison. As the growth curve for 10 Gy gamma-rays is similar to the growth curve for 5 Gy carbon ions, the RBE for carbon ion can be estimated to be about 2 for tumor growth control. This happens to be in the similar range observed for in vitro cell survival curves in Figure1. If mice are pretreated with 17AAG (three times before irradiation), tumor growth is further delayed. It seems that 17AAG itself caused some tumor growth inhibition, but its effect became more significant when combined with 10 Gy carbon irradiation. For some unknown reason, 17AAG plus 5 Gy carbon data may not show the distinct combination effect especially toward the later data points. This could mean that the combined treatment might be more effective with higher doses of carbon ions. Incidentally 1 day carbon treatment with a high dose has been used for some lung cancer patients at NIRS. After 20 Gy carbon irradiation, tumor growth was strongly controlled for a long period even without the drug (three of five mice showed complete response), while all irradiated mice showed no growth with pretreatment of 17AAG (five of five mice were completely controlled). Representative photographs showing tumor growth for various treatments are presented in Figure2B. The combined treatment resulted in the best tumor control. As an indicator for toxicity of the drug and irradiation, we measured the weight of all mice used in the study (Fig.3), but no significant differences were observed between treated and control mice, indicating that the treatment likely caused only minimal damage to their whole bodies.

### A cause of effective tumor control with 17AAG and carbon ion irradiation is homologous recombination repair inhibition by 17AAG pretreatment

**Figure 4:**
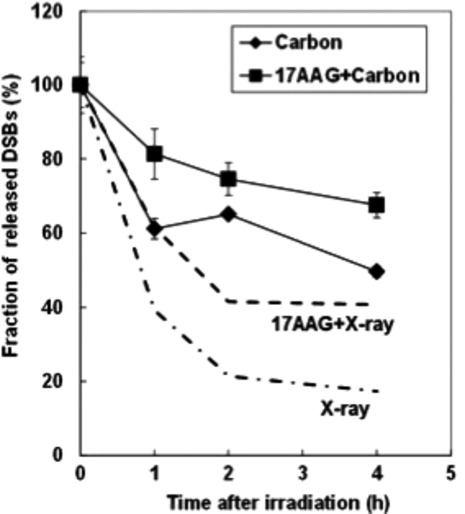
Comparison of DNA DSB rejoining kinetics in SQ5 cells irradiated with 20 Gy carbon ions with and without 17AAG pretreatment as measured by static gel-electrophoresis assay (Hirayama et al. 35). For comparison the DSB repair kinetics with X-rays are shown in dotted lines (Noguchi et al. 21). DSB, double strand breaks.

**Figure 5:**
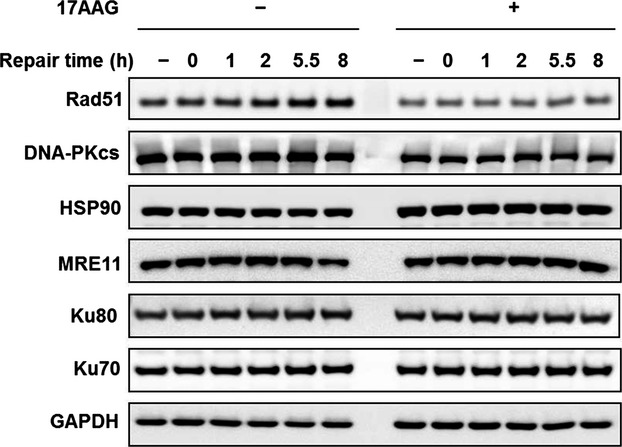
Immunoblotting evaluation of various proteins associated with DNA DSB repair. Results after carbon ion treatment with and without 17 AAG pretreatment as well as nonirradiated control are shown. GAPDH protein expression is a loading control. DSB, double strand breaks.

**Figure 6:**
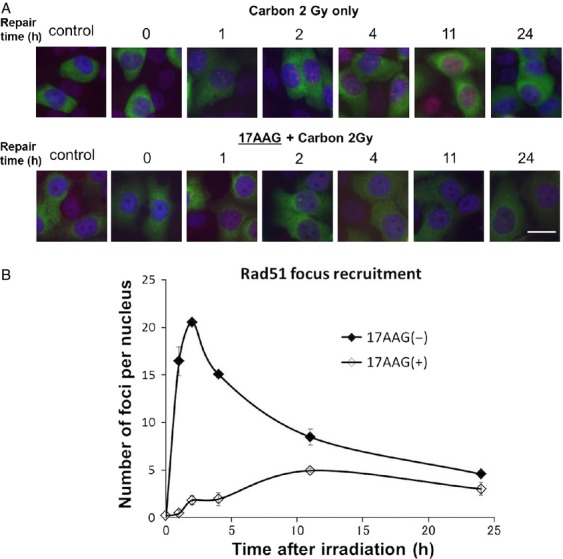
(A) Representative photographs of Rad51 foci appearance in carbon-irradiated SQ5 cells with and without 17 AAG pretreatment; cells were stained with both Rad51 and cyclin B antibodies to detect events associated with HRR. (B) Comparison of Rad51 foci appearance/disappearance kinetics obtained by counting dual stained foci in irradiated cells with and without 17AAG. HRR, homologous recombination repair.

In order to determine the mechanism of radiosensitization by 17AAG, we examined the repair of DNA DSBs in SQ5 cells irradiated with carbon ions by static field gel electrophoresis (SFGE) 35. Since we have shown that proteins associated with HRR were affected by 17AAG 21, and it was indicated that NHEJ pathway was mainly affected by high LET irradiation 5,37,38, we reasoned that inhibiting HRR pathway by 17AAG could further sensitize cells to high LET heavy-ion irradiation. Figure4 shows the DSB rejoining kinetics with 17AAG treatment and carbon ions as compared with carbon treatment alone. This result indicates that the cause of radiosensitization with heavy ions might be the inhibition of DSB repair by 17AAG as in the case with X-rays. Then we examined the expression of key proteins associated with DSB repair. As can be observed from Figure5, among all the proteins tested, only Rad51 was significantly reduced by 17AAG treatment and this seemed to be true both before and after carbon ion irradiation. There might have been a slight reduction in DNA-PKcs (NHEJ-associated protein) expression, but this subtle change might not have affected its functions; however, we still cannot exclude the possibility of NHEJ pathway involvement judging from our current data. In order to further study the detailed kinetics of Rad51 expression in carbon-irradiated cells, we performed immunofluorescence measurement with Rad51 antibody as well as cyclin B antibody to discriminate late-S/G2 phase-like cells. Figure6A shows examples of these double-stained samples and Figure6B shows the quantification of Rad51 foci counts where only cyclin B-stained cells were scored. Without 17AAG treatment, Rad51 foci show up quickly after carbon ion irradiation (top panel in Fig.6A), while the appearance of Rad51 foci was significantly delayed in 17AAG pretreated samples before carbon irradiation. These heavy-ion data suggest that HRR pathway is indeed affected by 17AAG pretreatment, as shown previously with the combined treatment with X-rays 21.

### G2 delay induced by carbon irradiation is further intensified by 17AAG pretreatment

**Figure 7:**
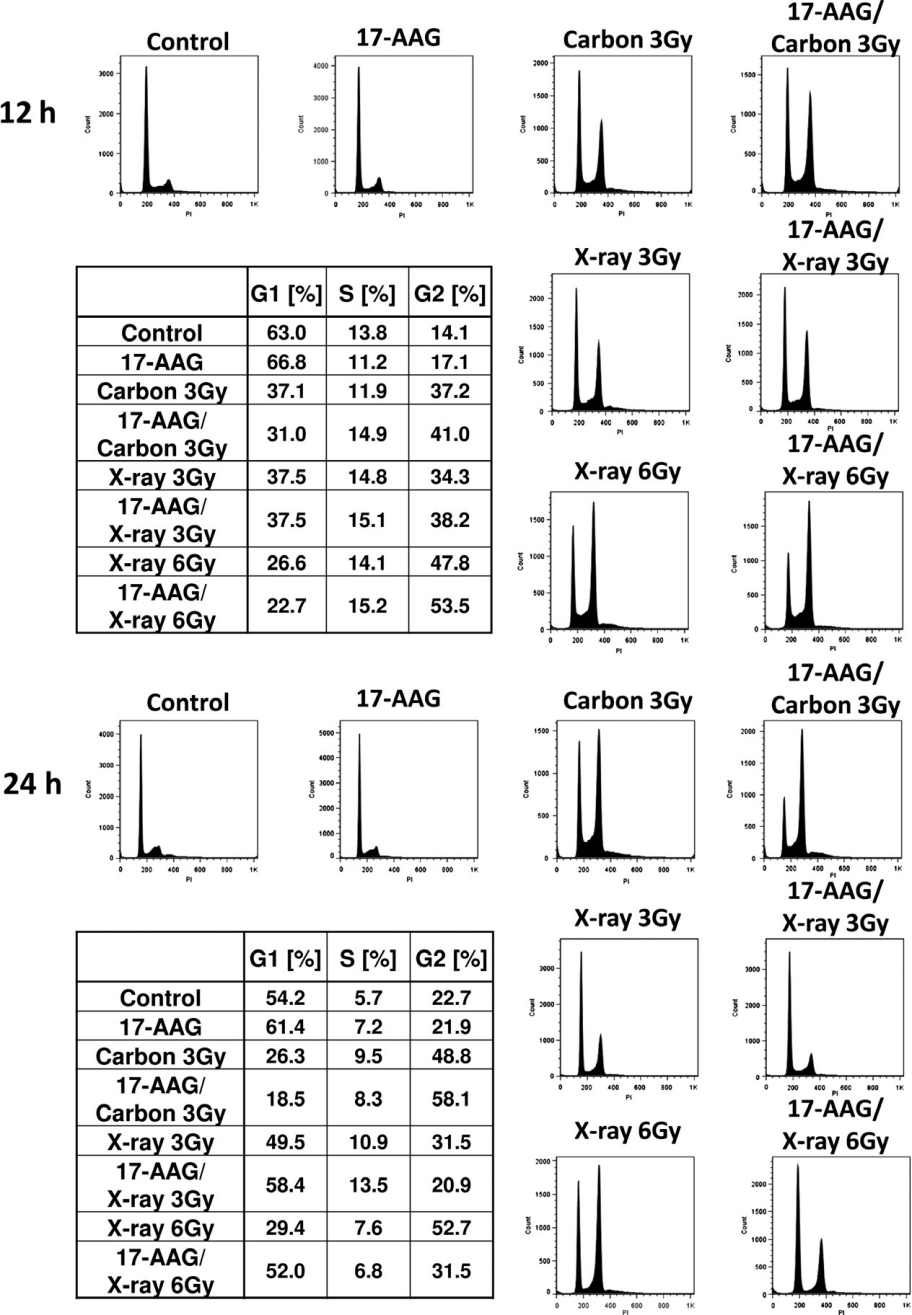
Flow cytometry analysis for cell cycle distribution in SQ5 cells irradiated with carbon or X-rays with/without 17 AAG pretreatment. Data of 12 and 24 postirradiation were provided for radiation doses 3 and 6 Gy. 3 Gy carbon ions may provide a similar cell survival level to 6 Gy X-rays (Equitoxic dose).

As Hsp90 inhibitor is known to affect the cell cycle checkpoint 23–29,32, we also studied cell cycle effects after various combinations of treatment with 17AAG and radiation. Figure7 shows our cell cycle data. The addition of 17AAG seemed to further intensify the radiation-induced G2 delay, and high LET carbon irradiation might induce even further delay than the same dose of X-rays (12 h data). At 24 h postirradiation, carbon 3 Gy data showed significant G2 delay, which was emphasized by the 17AAG treatment, while 3 Gy X-ray data seemed to show control-like cell cycle distribution, as most of the initial damage might have been resolved (repaired or processed) by this time. An interesting comparison could be made between 3 Gy carbon and 6 Gy X-ray data; we chose this dose to compare equitoxic samples, that is, carbon 3 Gy gives about the same cell survival level as 6 Gy X-rays. Although radiation only data with 3 Gy carbon and 6 Gy X-rays share a similar cell cycle profile, the addition of 17AAG leads to contrasting profiles; further G2 delay was observed in the carbon sample while G2 delay seemed to be resolved (control-like appearance) for the X-ray sample by this time.

## Discussion

In this report we have shown that the combination of Hsp90 inhibitor 17AAG and carbon ion irradiation provides good tumor control both in vitro and in vivo. As mentioned above, a number of reports have indicated radiosensitization effects by 17AAG or other Hsp90 inhibitors using gamma or X-rays 18–30, but radiosensitization by combining Hsp90 inhibitor and heavy-ion irradiation both in in vitro and in vivo has yet to be reported. Generally, it is thought that heavy-ion irradiation alone may provide sufficient tumor control and other additional factors might not be necessary. However, our results indicate that a better outcome could be obtained with the combination therapy when compared with carbon treatment alone. In fact, combination therapy with heavy ions might be more useful than combination therapy with X or gamma-rays, as tumors are physically targeted better with heavy ions and even higher cell killing at the tumor site can be expected with the combination therapy. Furthermore, as it was demonstrated that HRR-deficient cells manifested significant radiosensitization with high LET radiation 37,38, our sensitization data with 17AAG and high LET carbon radiation seem reasonable, since 17AAG was shown to inhibit HRR pathway with low LET irradiation 21, and similar inhibition also occurs with high LET radiation. As mentioned above, in line with this was a recent report that combining heavy-ion irradiation with microRNA targeting the HR pathway led to significant radiosensitization 34. Since it was indicated that among different DNA DSB repair pathways, high LET radiation mainly affects the nonhomologous end-joining (NHEJ) pathway, the combination of high LET heavy ions and HRR pathway inhibition would be a good choice for further effective tumor control. This was also demonstrated by our cell cycle study in Figure7. Further G2 delay was observed in cells with the combination treatment of 17AAG and heavy ions when compared with carbon irradiation alone, indicating that more DNA damage remains with this drug due to repair inhibition. More DNA damage is translated into more chromosome damage, leading to increased cell death after several attempted cell divisions after the combination treatment.

As heavy-ion irradiation may not always be available, the strategy described here could be applied to conventional photon or proton irradiation with Hsp90 inhibitor. In other words, instead of using heavy ions, another factor affecting NHEJ could be combined with an Hsp90 inhibitor; this could be different chemicals, heat or other agents. If a drug can control both NHEJ and HRR in irradiated cells, the combination treatment may be very efficient in killing tumor cells. A similar situation has been described using sulforaphane, a chemo-preventive agent, at a certain concentration resulting in radiosensitization by affecting both pathways 39. However, there seems to be no tumor selective effect with sulforaphane (our personal observation), so more exploration for the ideal agent is required.

The selective control of tumor cells with Hsp90 inhibitor with minimal effect on normal cells was repeatedly demonstrated in the past with radiation treatment 18,19,21, and our data with heavy-ion radiation (Fig.1B) also confirm this unique effect. In this regard, we are currently screening several other Hsp90 inhibitors with X-rays or carbon ions to determine which inhibitor(s) might demonstrate the most effective tumor control with low levels of toxicity. One note of caution must be raised, in that Hsp90 inhibitors may not work in all tumor types; some selective tumor types are more sensitive to these drugs 13. Further studies will be necessary to find out why this is the case. However, if Hsp90 inhibitors work as DNA repair inhibitors as shown in this and other reports, the combination therapy of Hsp90 inhibition and radiation might be applied to wider ranges of tumor types than the Hsp90 inhibitor treatment alone.

In summary, our results indicate that the combination of high LET heavy-ion irradiation and an Hsp90 inhibitor provides effective tumor growth delay. The cause of this effectiveness is likely to be the inhibition of two major DNA DSB repair pathways; heavy ions mainly reduce NHEH effectiveness and Hsp90 inhibitor decreases HRR efficiency, leading to good tumor control. This strategy needs to be tested with various other tumor types with the view of eventual clinical applications.
